# When Less Is More: Losing Genes on the Path to Becoming Human

**DOI:** 10.1371/journal.pbio.0040076

**Published:** 2006-02-14

**Authors:** Liza Gross

Students of human evolution got a big boost when the draft sequence of the chimp genome was published in 2005. Now their challenge is to comb through the combined 6 billion nucleotides for clues to the evolutionary forces that made humans odd man out in the primate family tree. Chimp and human DNA nucleotides differ by just 1.23%, sufficient genetic variation for natural selection to create a bipedal, big-brained primate lineage but small enough to suggest that every mutation has an evolutionary tale to tell.

Natural selection, it's typically thought, mostly favors “gain of function” mutations, which increase an organism's fitness while jettisoning “loss of function” mutations, which are considered deleterious. An alternative hypothesis proposes that gene loss—which can occur when mutations render functional genes inactive in a process called pseudogenization—can also confer selective advantage and promote evolutionary change.

In a new comparative analysis of the human and chimp genomes, Xiaoxia Wang, Wendy Grus, and Jianzhi Zhang find support for this “less is more” hypothesis. They analyzed 80 human pseudogenes—many involved in immunity and chemoreception—and show that positive selection explains how one pseudogene spread throughout human populations. Humans with the *CASP12* pseudogene, which retains its immune function in other mammals, are less susceptible to severe sepsis.

Several cases of gene loss have been proposed as adaptive opportunities for human evolution. To look for potentially momentous gene losses, Wang et al. turned to a human pseudogene database. Most of the 19,000 human pseudogenes in the database are “processed” pseudogenes, which derive from reverse-transcribed RNA that was reinserted into the genome, usually without the regulatory elements necessary for gene activation. Most processed pseudogenes never functioned. And nearly half of the 1,781 “nonprocessed” pseudogenes lack the mutations that would make them clearly nonfunctional. So the authors focused on the 887 “nonprocessed” pseudogenes that had the requisite loss-of-function mutations.

Comparing these human pseudogenes with the chimp genome flagged 83 sequences as closest matches—assumed to be chimp orthologs (derived from an ancestral gene during speciation). After ascertaining that the human pseudogenes have chimp orthologs and are truly nonprocessed pseudogenes, the authors whittled their list down to 67.

Most of the 80 pseudogenes studied (including 13 found in previous studies) play a role in immune response or chemoreception and belong to rapidly evolving gene families with species-specific members. Interestingly, mice lacking one of the pseudogenes, *Mbl1*, show higher resistance to sepsis and increased survival. Though this pseudogene likely spread through the human population too long ago to detect signs of selection (about 6–7 million years ago), the authors reasoned that if mice gained an advantage from losing an immunity gene, humans might, too.

To test this possibility, they focused on another sepsis-related pseudogene, *CASPASE12 (CASP12)* that is still undergoing pseudogenization. In the nonfunctional (null) allele, a mutation replaces a C with a T nucleotide (called a C/T polymorphism), producing truncated, nonfunctional CASP12 proteins. The null T allele is linked to sepsis resistance in humans. All non-Africans (that is, individuals of Asian or European descent) carry two copies of the T allele, which occurs with 89% frequency in individuals of African descent.

The authors used population genetic techniques to test whether positive selection drove the high frequency of the T allele. If it did, sequences surrounding the T allele should have lower variation than those flanking the C allele. And that's just what the authors found. While the T allele existed at a much higher frequency than the C allele in the sampled populations, it had far fewer polymorphisms across nine noncoding regions adjacent to the C/T polymorphism.

Wang et al. modeled different demographic scenarios and mutation rate estimates using the polymorphism data to explain the source of the observed low diversity. Their simulation results “strongly suggest” that positive selection drove the spread of the T allele. Since non-Africans and Africans carry identical T alleles, the null mutations likely share a common ancestor, originating sometime before modern humans left Africa 40,000–60,000 years ago. Assuming that selection acted on the variant as soon as it appeared, the allele would need about 51,000–55,000 years to reach its current frequency, suggesting that the adaptive pseudogenization started shortly before the African emigration.

These findings show that gene loss provides not just the opportunities but the means for selection to act. Since pseudogenization in vertebrate and other eukaryotic genomes is common, the authors speculate that adaptive gene loss may be as well. As the chimp genome sequence becomes more refined, researchers will have fresh opportunities to explore how gene loss shaped the unique traits that define human biology—and formulate new hypotheses to test the breadth of a “less is more” strategy in evolutionary change.

## 

**Figure:**
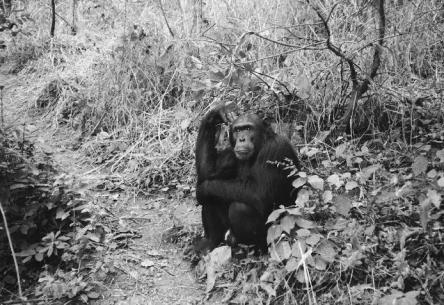
Analyzing "pseudogenes" that lost their functionality since humans and chimps diverged over 6 million years ago promises to shed light on the genetic basis of human origins. (Photo: Curt Busse, Gombe National Park, Tanzania)

